# Birth Order and Screen Time as Predictors of Emotion Comprehension Development in 5– to 7–Year-Old Boys and Girls

**DOI:** 10.11621/pir.2026.0204

**Published:** 2026-06-30

**Authors:** Elena A. Chichinina, Olga V. Almazova

**Affiliations:** a Federal Scientific Center for Psychological and Interdisciplinary Research, Moscow, Russia; b Lomonosov Moscow State University, Russia

**Keywords:** emotion comprehension, birth order, active screen time, passive screen time, preschool children

## Abstract

**Background.:**

Early childhood is a sensitive period for the development of emotion comprehension. Although family factors are known to influence emotion comprehension, the specific role of birth order remains understudied. In the modern context, research on predictors of emotion comprehension should control for screen time.

**Objective.:**

This study investigates whether birth order predicts emotion comprehension development in 5–6-year-old children over a one-year period, controlling for screen time.

**Design.:**

The sample consisted of 84 boys and 83 girls recruited from two-child families with age gap between siblings not more than 5 years. During the study’s first stage (T1), the children were in the penultimate year of kindergarten. The study’s second stage was one year later. The Test of Emotion Comprehension (TEC) was used to evaluate emotion comprehension. Data on birth order, age difference between siblings, and screen time were collected via a questionnaire for caregivers.

**Results.:**

Birth order was not a significant predictor of the emotion comprehension development in boys and girls. However, in the exploratory model for girls, higher active screen time at T1 was associated with smaller gains in emotion comprehension over one year.

**Conclusion.:**

The findings are exploratory and require further testing; future research should formally test whether sex moderates the association between active screen time and emotion comprehension development. The absence of birth order effects may reflect insufficient statistical power rather than a true null relationship. Subsequent studies with larger samples should examine why active screen time affects emotion comprehension development in girls.

## Introduction

Early childhood is a sensitive period for the development of emotion comprehension (Pons et al., 2004). This capacity is a well-established predictor of subsequent social competence as well as academic achievement (Guseva et al., 2025; Pavlova, 2025; Richard et al., 2025). Therefore, investigating factors that influence its development in early childhood is essential. The emotion comprehension development in children is shaped by an interplay of biological, genetic, cultural, and environmental aspects, with the family context playing a critical role (Buss et al., 2019; Guseva, 2025; Kamalova, 2024). Research has already demonstrated broad family influences, particularly parenting styles, on children’s emotional development (Quang Dao & Le, 2025; Al-Elaimat et al., 2020). However, the specific role of a child’s birth order in predicting individual differences in emotion comprehension is not researched thoroughly (Aslanova et al., 2024; Dunn, 2015). The present study aims to address this gap by investigating birth order as a predictor of emotion comprehension development. Additionally, research on emotion comprehension predictors in contemporary context should control for screen time, given its substantial impact on children’s emotional development (Jia et al., 2025; Kiseleva et al., 2024; Skalická et al., 2019). Although birth order is not a modifiable factor, understanding its potential effects is important for tailoring parenting strategies (Black et al., 2026). Understanding the birth-order effects is important for enabling parents to tailor their parenting strategies to each child’s position in the family. Understanding the screen-time effects is also crucial, given that caregivers can influence their children’s screen time.

### Emotion Comprehension in Children

Emotion comprehension is a cognitive ability that involves understanding the nature, causes, consequences, and expression of emotions in oneself and others (Pons et al., 2004). The predominant theoretical framework in this domain is the Three-Component Model proposed by Pons et al. (2004). This model introduces a hierarchical developmental trajectory wherein children’s emotion comprehension progresses through three successively developing components. The External Component (typically emerging between the ages of 3 and 5) involves comprehension based on perceptible, external, situational cues, such as recognizing facial expressions or simple situational causes of emotions. The Mental Component (develops between the ages of 4 and 6) incorporates an understanding of the role of internal mental states including beliefs, desires, and memories as emotions’ mediators. The Reflective Component (develops from the age of 6–8) entails complex reflection, such as managing or hiding emotions, feeling mixed and moral emotions. Thus, examining emotion comprehension at the ages of 5–7 years is most informative, as this ability is already partially formed, yet not fully developed. This implies that any interventions aimed at fostering emotion comprehension would be particularly relevant at this stage.

### Birth Order and Emotion Comprehension Development in Children

During early childhood, children’s primary social interactions occur with parents and siblings, with the sibling relationship constituting a particularly significant sociocultural context as children often spend comparable or greater time with siblings than with parents (Dunn, 2015; Hill & Palacios, 2019; Oshchepkova et al., 2025). While numerous studies have explored the link between birth order and children’s emotional development (Kramer, 2014; Villanueva Iglesias & García-Martín, 2023), research targeting its specific association with emotion comprehension remains insufficient. This gap calls for a theoretical examination of potential mechanisms. One perspective suggests that first-born children may develop superior emotion comprehension due to exclusive early access to parental attention, communication, and guided play, since these factors are crucial for emotion comprehension development (Blake, 1981; Kim & Wang, 2023). Conversely, a competing hypothesis emphasizes an advantage for later-born children, who can observe and learn from an older sibling and potentially benefit from emotional skill scaffolding during sibling interactions (Rolan et al., 2018). Supporting this, one study with adult participants found higher emotional intelligence among those born in the middle or last in the family (Villanueva Iglesias & García-Martín, 2023). However, other scientific evidence suggests that birth order has little to no substantive relation to personality trait development (Damian & Roberts, 2015), which may extend to emotion comprehension. Therefore, the effect of birth order on emotion comprehension is a largely debated issue.

In addition to birth order, the age gap between siblings may play an important role in children’s emotion comprehension development. Several studies have examined the association between the age gap between siblings and children’s emotional development (Dhamrait et al., 2022; Whiteman et al., 2011). Siblings with a small age gap between them may be more likely to engage with each other as peers. Consequently, these siblings may benefit more from inter-sibling interactions compared to those with a larger age gap. In contrast, siblings with a large age gap may have minimal interaction, or the older sibling may assume a parent-like role toward the younger one. Thus, when examining birth-order effects, it is important to also control for the age gap between siblings.

### Screen Time and Emotion Comprehension Development in Children

Excessive screen time in early childhood is a known risk factor for adverse socio-emotional outcomes, including deficits in emotion comprehension. Evidence indicates that greater screen time predicts increased emotional dysregulation, socio-emotional delays and reduced emotional reciprocity with caregivers (Jia et al., 2025; Skalická et al., 2019). Furthermore, a child’s screen time often correlates with parental screen time (Ishii et al., 2022), creating a situation when high parental screen time leads to “phubbing” (phone-snubbing). This behavior displaces the high-quality, live parent-child interaction fundamental for development of emotion comprehension (Leontiev, 1975). From a sociocultural perspective (Zinchenko et al., 2024), emotions are culturally mediated psychological tools that children internalize through guided social interaction. This process requires real-time engagement to connect facial expressions and situational contexts with emotional states. Digital content typically fails to provide this crucial experience for two primary reasons. Firstly, the media content commonly used by preschoolers often lack authentic depictions of human faces and the nuanced complexity of real emotions. Secondly, the development of emotion comprehension depends on live interactive feedback, which allows a child to validate his/her interpretations of others’ emotions and better understand his/her own. Therefore, prolonged screen time hinders emotion comprehension by displacing essential live interaction and impoverishing family communication (Jia et al., 2025).

However, the association between screen time and emotion comprehension is not uniform; it is moderated by both sex and the type of screen time (active vs. passive). Skalická et al. (2019) found sex-specific patterns: passive TV viewing showed a stronger negative association with emotion understanding in girls, whereas active digital gaming was linked to lower levels of emotional understanding in boys. There are two types of screen time: such as active screen time (*i.e.* time spent interactively using digital devices, such as digital games and educational apps) and passive screen time (*i.e.* time spent watching video content) (Lakicevic et al., 2025; Liao & Tian, 2025; Nustad & Abrahamsson, 2026). Moreover, according to empirical data, the first type may be more productive for child development (Liao & Tian, 2025; Nustad & Abrahamsson, 2026). For instance, interactive educational platforms provide enriching experiences that may support the development of emotion comprehension (Gou & Perceval, 2023). Therefore, research focusing on the link between screen time and emotion comprehension must distinguish between passive and active screen time and should analyze data considering potential sex differences.

In research, it is pivotal to differentiate between passive and active screen time, however, in the structure of both passive and active screen time, a rapidly evolving universe of options is possible. Thus, within the passive screen time, there is a spectrum of activities ranging from concentrated family viewing of a film to scrolling reels. And within the active screen time, there is a selection of activities varying from offline games and applications with very simple mechanics and without interaction with other people to multiplayer online games and various games and applications using artificial intelligence, extended, or augmented, or virtual reality (Blumberg et al., 2024; Uǧ̈raş et al., 2025). Currently there is no empirical basis showing how different new variants of passive and active screen time relate to children’s development and learning (Blumberg et al., 2024). However, it’s possible to identify certain principles that screen time should meet to contribute to children’s development, including emotion comprehension development. And, first and foremost, only active screen time can satisfy these principles. Firstly, it is crucial that games and apps are developed with the participation of researchers and are not exclusively commercial games (Flynn et al., 2021). Secondly, feedback, collaboration, and communication are vital when playing games or using apps (Blumberg et al., 2024). Thirdly, the game or app should facilitate the skill transfer to reality (*i.e.* far transfer) (Pasqualotto et al., 2023).

### Current Research

Despite recognition of birth order as a potential contributor to individual differences in emotion comprehension, empirical evidence directly examining this association remains scarce (Aslanova et al., 2024; McAlister & Peterson, 2013). Also, in the context of digitalization, screen time may affect the development of emotion comprehension (Gou & Perceval, 2023). Accordingly, the aim of this study was to investigate contributions of birth order and screen time (passive vs. active) to the emotion comprehension development in preschool children over a one-year period. We asked ourselves the following research question: Is birth order a predictor of emotion comprehension development in 5–6-year-old children over a one-year period, when controlling for screen time? All children were from two-child families with a sibling age gap not exceeding five years.

This choice of demographics was guided by the following considerations. First of all, this family configuration is the most prevalent among Russian families with more than one child (Sukneva et al., 2020). Secondly, the emotion comprehension development is particularly pronounced in the years immediately preceding school entry, as this ability is foundational for school adaptation and subsequent socio-emotional functioning (Richard et al., 2025). Thirdly, to ensure developmental comparability within sibling dyads, the age difference between the first-born and second-born child was restricted to a maximum of five years – a criterion symmetrically applied to both older and younger siblings.

It’s also essential to note the study’s several methodological features. First, in Russia, children begin formal schooling at the age of seven. Prior to elementary school, they spend most weekdays in kindergarten, where digital devices are typically not available. This makes it important to examine weekday and weekend screen time separately. Second, boys and girls are shaped by distinct biological and contextual influences that affect their cognitive, motor, social, and emotional development (Beren-baum et al., 2007; Mengxia, 2024; Sobkin & Skobeltsina, 2015). These sex differences, together with gendered parenting practices, may therefore lead to variations in behavior, screen time, and emotion comprehension development between boys and girls of different birth orders (Berenbaum et al., 2007; Mengxia, 2024; Sobkin & Skobeltsina, 2015). Consequently, we adopted a sex-stratified approach in this research, comparing boys with boys and girls with girls. Third, we used questionnaires for caregivers to collect information on children’s screen time. Although this method does not ensure complete validity, it represents the most feasible approach for older preschool-age children, as two alternative methods are unavailable for 5– to 6–year-olds. Directly asking children about their screen time is not possible, as children of this age cannot accurately estimate time. Furthermore, obtaining objective screen time statistics from digital devices is also not feasible, because 5– to 6–year-old children do not yet possess their own digital devices and instead use their parents’ devices. In addition, collecting objective screen time data from caregivers’ devices would violate their privacy. Nevertheless, the exclusive reliance on caregivers’ reports introduces potential recall and social desirability biases, which may have inflated associations or skewed the differentiation between active and passive screen use. Therefore, any finding derived from this subjective measure — including the primary novel result of this study — must be interpreted with caution.

## Methods

### Participants

The sample consisted of 167 children recruited from municipal kindergartens in three regions of Russia. Their primary caregivers also participated in the study. Most children came from families of middle socioeconomic status: 90% of mothers had higher education, and 80% of families reported a middle level of income.

There were the following inclusion criteria for the children: (a) The absolute age difference between the siblings was not greater than five years; (b) Children were from families with two children; (c) Children demonstrated neurotypical development, and (d) were native speakers of Russian.

Out of the 167 children, 84 were boys (50% of whom were second-born) and 83 were girls (61% of whom were second-born). During the study’s first stage (T1), the children were in the penultimate year of kindergarten (their age was *M* = 70.3 months, *SD* = 4.2). During the study’s second stage (T2), the children were one year older, and they were in the last year of kindergarten (their age was *M* = 81.2 months, *SD* = 4.0).

### Materials

Emotion comprehension was assessed via the *Test of Emotion Comprehension (TEC)* (Pons & Harris, 2000). The TEC is presented as a picture book (separate versions for girls and boys), where the examiner reads short, contextual stories accompanied by illustrations. After each piece, the child is asked to indicate how the protagonist feels by pointing to one of four schematic facial expressions (such as happy, sad, angry, scared). The total score ranges from 0 to 9, with higher scores indicating greater emotion comprehension. A change score in emotion comprehension over a year (delta) was computed by subtracting the T1 score from the T2 score.

Data on birth order and age difference between siblings were collected via a questionnaire for caregivers, as they were asked to write the child’s date of birth and sex, age and sex of each sibling if present. Then only the children from two-child families with absolute difference in age between siblings not more than 5 years were included in the sample.

Data on screen time were collected with the same questionnaire. Caregivers were asked to write the number of hours and minutes that the child usually spends watching cartoons and videos (passive screen time) separately on an average weekday and weekend day. In the same manner, caregivers were asked to share the number of hours and minutes that the child usually spends on the computer, tablet, smartphone, game console, not counting the time spent watching cartoons and videos (active screen time) separately on an average weekday and weekend day. Then active and passive weekly screen time in minutes was calculated.

Several important methodological considerations regarding the screen time data collection should be mentioned. First of all, it is pivotal to collect screen time data for weekdays and weekends separately, as screen time is known to differ substantially between these types of days (Lakicevic et al., 2025). Second, although relying solely on caregivers’ reports introduces significant recall and social desirability biases, this method has proven effective in previous studies (Veraksa et al., 2021), and we acknowledge this limitation in the interpretation of our findings.

Data on the level of family income and maternal education were collected via the same questionnaire. These data were used to describe the sample. Caregivers were asked: “What is your family income?” with the answer options: “below average”, “average”, “above average”, “other”. They were also asked to report the educational level of the child’s mother.

### Procedure

The emotion comprehension assessment procedure during both T1 and T2 was identical. The test was administered in a quiet room by a trained researcher with each child individually in the morning, between 8 and 11 am. The assessment required approximately 10 min per child. At T1 and T2, after the emotion comprehension assessment, the caregivers received an email with the questionnaire.

This study was conducted in accordance with the Declaration of Helsinki ethical standards. This research was also approved by the Ethics Committee of scientific research of the Federal Scientific Center of Psychological and Multidisciplinary Research (approval + 4, dated 19.12.2025). This study involved both adult participants (caregivers) and their children. Written informed consents were obtained from all caregivers for their own participation, as well as for the participation of their children. Additionally, each child consented to participate as well. The study procedures were explained in an age-appropriate manner to the children prior to obtaining their consents. Caregivers were informed about the purpose of the research, the confidential handling of their data, and the right to withdraw from the study at any time without consequences or explanations. To protect participants’ privacy, all identifying information was replaced with unique participant codes prior to analysis.

### Statistical Analysis

All analyses were performed using Jamovi version 2.0.0.0. The significance level (α) was set at .05 for all tests.

At first the descriptive statistics (mean, SD, min, max) were reported. Also, the normality of distribution for all continuous variables (child’s age, screen time measures, age difference, emotion comprehension scores at T1, and emotion comprehension change) was assessed using the Shapiro-Wilk *W* test.

Then first-born and second-born children were compared on baseline characteristics and outcome variables within each sex. As most variables violated the assumption of normality, to compare first-born and second-born children, non-parametric Mann-Whitney *U* test was used. The rank-biserial correlation (*r_b_*) was calculated as a measure of effect size for these comparisons. This comparison, if any differences are absent, justifies the subsequent inclusion of birth order as a predictor in the regression models without concern for major confounding by these variables.

After that Spearman’s correlations between screen time at T1 and T2 in boys and girls were calculated to examine if it was possible to include screen time at T2 as predictor in further General Linear Models*.* Cut-out points for Spearman’s correlation coefficient were as follows: .09 < *r* < .20 weak, .19 < *r* < .30 moderate, V ≥ .30 strong (López-Martín & Ardura-Martínez, 2023).

Then separate General Linear Models were constructed for boys and girls to identify the unique contribution of predictors to the one-year change in emotion comprehension. Five predictors were simultaneously entered as independent variables: birth order (dichotomous: first-born, second-born), weekly passive screen time at T1 (continuous, in minutes), weekly active screen time at T1 (continuous, in minutes), absolute age difference between siblings (continuous, in years), baseline emotion comprehension score at T1 (continuous). Model assumptions (such as linearity, homogeneity of variances, independence, and normality of residuals) were checked and deemed satisfactory. The overall significance of each model was evaluated via the omnibus *F*-test. For significant models, the unique contribution of each predictor was assessed based on its *t*-statistic, *p*-value, and partial eta-squared (η^2^) as a measure of effect size. Unstandardized regression coefficients (*B*) with their standard errors (*SE*) and 95% confidence intervals (*CI*) are reported. The model’s explanatory power is indicated by the adjusted coefficient of determination *R^2^*.

Finally, we estimated the statistical power of the main regression analyses given the observed sample sizes (boys and girls). A post hoc power analysis was performed via G*Power version 3.1.9.4. (*F* tests, linear multiple regression: Fixed model, *R^2^* deviation from zero) (Faul et al., 2007). We calculated the achieved power to detect a medium effect size (*f^2^*= .15) with five predictors at α error probability = .05. The analysis revealed a power (1 - β error probability) of .76 for the boys’ model (*n* = 84) and .77 for the girls’ model (*n* = 83). Thus, the achieved power was slightly below the conventional threshold of .80, suggesting a marginally increased risk of Type II error (Gupta et al., 2016).

## Results

*Table 1* shows descriptive statistics for key study variables in boys and girls. Most variables were not normally distributed; therefore, the non-parametric Mann-Whitney *U* test was used for subsequent analyses.

**Table 1:** Descriptive Statistics in Child’s Age, Active and Passive Screen Time, Absolute Difference in Age between Siblings, Emotion Comprehension at T1, and Emotion Comprehension Change in Boys and Girls

	Boys				Girls			
	M	SD	Min; Max	W; p	M	SD	Min; Max	W; p
Age, months, T1	71.9	3.8	63; 84	.980; .206	72.3	3.6	64; 80	.980; .217
Weekly minutes, passive T1 screen time,	585	301	75; 1380	.961; .012	620	389	60; 1920	.924; < .001
Weekly minutes, active T1 screen time,	233	258	0; 1500	.791; < .001	162	188	0; 840	.816; < .001
Weekly minutes, passive T2 screen time,	688	404	0; 1740	.954; .042	589	331	0; 1620	.942; .008
Weekly minutes, active T2 screen time,	365	360	0; 1605	.841; < .001	371	850	0; 1640	.345; < .001
Age siblings, difference |years| between	2.8	1.1	1; 5	.900; < .001	3.1	1.2	1; 5	.913; < .001
Emotion T1 comprehension,	4.9	1.3	2; 8	.944; .001	5.1	1.3	2; 8	.939; < .001
Emotion delta comprehension,	1.4	1.8	–3; 5	.965; .151	.8	1.7	–3; 4	.950; .003

*Table 2* demonstrates comparisons for key study variables between first-born and second-born children. For boys and for girls, there were no statistically significant differences between first-born and second-born children on any of the measured variables. These preliminary analyses confirm that the groups defined by birth order were well-matched at baseline (T1) within each sex.

**Table 2:** Medians, IQR and Differences in Child’s Age, Active and Passive Screen Time, Absolute Difference in Age between Siblings, Emotion Comprehension at T1, and Emotion Comprehension Change in Boys and Girls with Different Birth Order

	First-born children = 3		Second-born children =1		Differences		
	Median	IQR	Median	IQR	U	p	r_b_
	**Boys**						
Age, months, T1	72	4	71	4	741	.207	.160
Weekly passive screen time, minutes, T1	540	428	570	458	816	.554	.075
Weekly active screen time, minutes, T1	158	270	228	368	722	.146	.182
Weekly passive screen time, minutes, T2	600	560	660	580	316	.693	.065
Weekly active screen time, minutes, T2	290	311	210	416	268	.391	.143
Age difference between siblings, |years|	3	1	2.5	2	853	.787	.033
Emotion comprehension, T1	5	2	5	2	789	.394	.105
Emotion comprehension, delta	1	2	1	2	814	.535	.078
	**Girls**						
Age, months, T1	72	6	73	4	723	.385	.114
Weekly passive screen time, minutes, T1	510	543	540	290	807	.933	.012
Weekly active screen time, minutes, T1	65	210	120	240	706	.294	.135
Weekly passive screen time, minutes, T2	540	360	560	510	402	.869	.027
Weekly active screen time, minutes, T2	123	278	240	345	230	.185	.227
Age difference between siblings, |years|	3	2	3	2	782	.743	.042
Emotion comprehension, T1	5	2	5	2	816	1.000	< .001
Emotion comprehension, delta	1	2	1	2	803	.905	.016

*Table 3* presents Spearman’s correlations between screen time at T1 and T2 in boys and girls. For boys as well as for girls, passive screen time at T1 and T2 were strongly positively correlated, and active screen time at T1 and T2 were strongly positively correlated as well. In this regard, screen time at T2 was not included as a predictor in the General Linear Models. Moreover, the analysis of the delayed effects of screen time on emotion comprehension development was of the greatest interest.

**Table 3:** Spearman’s Correlations between Screen Time at T1 and T2 in Boys and Girls

	Weekly passive screen time, T1	Weekly active screen time, T1	Weekly passive screen time, T2	Weekly active screen time, T2
**Boys**				
Weekly passive screen time, T1				
Weekly active screen time, T1	*r* = .332; *p* = .002			
Weekly passive screen time, T2	*r* = .730; *p* < .001	*r* = .291; *p* = .036		
Weekly active screen time, T2	*r* = .186; *p* = .196	*r* = .429; *p* = .002	*r* = .274; *p* = .054	
**Girls**				
Weekly passive screen time, T1				
Weekly active screen time, T1	*r* = .226; *p* = .040			
Weekly passive screen time, T2	*r* = .654; *p* < .001	*r* = .283; *p* = .031		
Weekly active screen time, T2	*r* = .044; *p* = .757	*r* = .482; *p* < .001	*r* = .251; *p* = .079	

*Table 4* shows separate General Linear Models for boys and girls. So, speaking of boys, the overall model was statistically significant, accounting for approximately 36% of the variance in one-year emotion comprehension change. The baseline level of emotion comprehension at T1 was the only significant predictor in the model. The negative regression coefficient (*B* = -.876) indicates a compensatory pattern: boys with higher initial emotion comprehension at T1 showed a smaller gain over the following year compared to boys with lower initial scores. None of the other predictors foretold significantly the change in emotion comprehension for boys.

**Table 4:** General Linear Models for Emotion Comprehension Change Over a Year Based on Birth Order, Active and Passive Screen Time at T1, Absolute Difference in Age Between Siblings, and Emotion Comprehension at T1

	*B (SE)*	Upper; 95% Lower *CI*	*t*	*p*	η^2^
**Boys**					
Intercept	1.155 (.153)	.850; 1.459	7.547	< .001	
Birth order	–.102 (.310)	–.719; .515	–.328	.744	.001
Weekly passive screen time, T1	< –.001 (< .001)	–.001; < .001	–.505	.615	.003
Weekly active screen time, T1	< .001 (< .001)	–.001; .001	.176	.861	.000
Age difference between siblings	.222 (.143)	–.063; .506	1.550	.125	.030
Emotion comprehension, T1	–.876 (.126)	–1.127; –.625	–6.939	< .001	.382
Model info: *F* (5; 78) = 10.329; *p* < .001, η^2^= .398, adj. *R^2^*= .360					
**Girls**					
Intercept	.825 (.142)	.543; 1.107	5.825	< .001	
Birth order	–.164 (.289)	–.740; .412	–.566	.573	.004
Weekly passive screen time, T1	< .001 (< .001)	< –.001; < .001	.557	.579	.004
Weekly active screen time, T1	–.002 (< .001)	–.003; < .001	–2.006	.048	.050
Age difference between siblings	–.006 (.117)	–.238; .227	–.048	.962	.000
Emotion comprehension, T1	–.771 (.109)	–.987; –.555	–7.104	< .001	.396
Model info: *F* (5; 77) = 13.462; *p* < .001, η^2^= .466, adj*. R^2^*= .432					

For girls, the overall model was also significant and explained a larger portion of the variance than in boys – approximately 43%. As with boys, the strongest and most significant predictor was the baseline emotion comprehension score at T1, showing a similar compensatory effect. However, for girls, weekly active screen time at T1 emerged as an additional significant predictor. The negative coefficient (*B* = -.002) suggests that girls who spent more active screen time at T1 demonstrated a slightly smaller gain in emotion comprehension over the subsequent year. Birth order, weekly passive screen time, and the age difference between siblings were not significant predictors for girls.

## Discussion

Preschool years represent a period of active emotion comprehension development (Pons et al., 2004), shaped by a range of family and environmental influences (Buss et al., 2019; Guseva, 2025). Among these, birth order and screen time have emerged as two salient but rarely examined predictors. The present study aimed to determine whether birth order predicts the one-year development of emotion comprehension in 5– to 6–year-old boys and girls after controlling for screen time.

Birth order was not a significant predictor of the one-year development of emotion comprehension. A post-hoc power analysis revealed that the achieved statistical power for the regression was .76, which falls below the conventional threshold of .80. This suggests that our study may have been underpowered to detect small-to-medium effects for this variable. Consequently, the null findings do not conclusively demonstrate the absence of a relationship; rather, they may reflect Type II errors. Future studies with larger samples are needed to definitively rule out birth order as a potential influence on emotion comprehension development. These considerations also apply to the age gap between siblings and passive screen time, which similarly did not predict the one-year development of emotion comprehension.

However, if birth order is indeed not a significant predictor of the one-year emotion comprehension development and this finding is not attributable to insufficient statistical power, then we have to consider several things. Existing research presents conflicting evidence regarding the effect of birth order on emotion comprehension (Damian & Roberts, 2015; Kim & Wang, 2023; Rolan et al., 2018). One possible explanation is that the family resources availability (material, social, emotional, and time allocation) may play a more decisive role than birth order per se. Nevertheless, the lack of significant associations for birth order in our models should be interpreted with caution.

The null finding for birth order, though qualified by limited power, suggests that screen time can be examined without major confounding by birth order in this sample*.* In our sample, screen time did not differ significantly between first-born and second-born children, further supporting the independence of these two predictors. Thus, while birth order remains a theoretically relevant family characteristic, screen time represents a distinct and potentially more modifiable daily activity that may have direct implications for emotion comprehension development. We now turn to the screen time findings, which constitute the primary focus of the remainder of the discussion.

For girls, active screen time at age 5–6 was negatively associated with emotion comprehension gains. The mechanisms underlying this association remain unclear, as we did not directly measure potential mediators. One possible explanation, requiring direct testing, is that active screen time may displace forms of non-digital play that support socio-emotional development (Smits-van der Nat et al., 2024). However, other pathways – such as the specific content of digital games (Plotnikova et al., 2023; Blumberg et al., 2024; Chu et al., 2024), level of imagination (Francis & Gibson, 2023), or quality of parent–child interactions (Wei et al., 2025) – could also be involved, but these remain speculative and should be examined in future studies with appropriate measures.

For boys, active screen time was not a significant predictor. One hypothesis, requiring direct testing, is that the types of play and digital activities typical for boys may be less closely tied to emotion comprehension, so their displacement might have a weaker effect. This interpretation is tentative and needs empirical verification.

Screen time did not differ between first-born and second-born children from two-child families. This absence of differences allowed us to include screen time as a predictor in our models. However, some studies have discovered that screen time is higher in second-born children (Black et al., 2026; Chichinina et al., 2025). Black et al. (2026) reported that increases in time spent on digital media and corresponding reductions in time spent on enrichment activities are generally greater for 10- to 14-year-old children. Chichinina et al. (2025) found that among 5– to 6–year-old kids, only active screen time was higher in second-born children, whereas passive screen time did not vary between children with different birth orders. Thus, differences in screen time among older preschool-aged children may be relatively subtle. Moreover, the present study’s null findings may also be attributed to the relatively small sample sizes.

## Conclusion

The present study examined whether birth order and screen time predict one-year emotion comprehension development in 5- to 6-year-old children. Birth order was not a significant predictor, although the study was underpowered to detect small-to-medium effects; larger samples are needed to draw firm conclusions. Active screen time negatively predicted emotion comprehension development in girls. Passive screen time showed no effects in either sex. However, these conclusions are tempered by methodological limitations. The null findings for birth order, in particular, must be interpreted with considerable caution, as post-hoc power analyses revealed statistical power below the conventional .80 threshold. This may suggest that the absence of significant effects reflects Type II error rather than a true lack of association. Future research with adequately powered samples should formally test whether sex moderates the association between active screen time and emotion comprehension development, and, if moderation is confirmed, explore the underlying mechanisms (*e.g.*, play displacement, parent–child interaction quality). Until such studies are conducted, the question of whether birth order influences emotion comprehension development remains open. Also, future research requires a more detailed analysis of the screen time structure, and not just a separate passive and active screen time consideration.

## Limitations

This study carries several limitations. To begin with, the post-hoc power analysis revealed that statistical power fell below the conventional threshold of .80, with values of .76 for the boys’ model and .77 for the girls’ model. Therefore, the absence of significant birth order effects on the one-year development of emotion comprehension may be attributed to insufficient statistical power rather than a true lack of association. Second, screen time was assessed via maternal report, a measure that can be influenced by inaccurate recall and tendencies toward socially desirable answers. It is possible that, in the absence of such bias, additional effects might have emerged: specifically, an effect of active screen time in boys (rather than in girls only) and an effect of passive screen time (rather than active screen time only). Third, the study used separate regression models for boys and girls without formally testing the Sex × Active Screen Time interaction. Therefore, the observed sex-related differences should be interpreted as exploratory rather than confirmatory.
